# Target-Controlled Infusion of Propofol: A Systematic Review of Recent Results

**DOI:** 10.1007/s10916-025-02187-y

**Published:** 2025-04-28

**Authors:** Pavla Šafránková, Jan Bruthans

**Affiliations:** 1 Department of Biomedical Technology, Faculty of Biomedical Engineering, Czech Technical University in Prague, nám. Sítná 3105, Kladno, CZ-272 01 Czech Republic; 2 Department of Anesthesiology and Intensive Care, General University Hospital, U Nemocnice 499/2, Prague, CZ-128 08 Czech Republic

**Keywords:** Target-controlled infusion, Propofol, Pharmacokinetics, Mathematical modeling in anesthesia

## Abstract

This study presents a systematic review conducted according to the PRISMA 2020 guidelines, evaluating pharmacokinetic-pharmacodynamic (PK-PD) models for target-controlled infusion (TCI) of propofol. A structured search was performed across PubMed, Summon, Google Scholar, Web of Science, and Scopus, identifying 427 sources, of which 17 met the inclusion criteria. The analysis revealed that nine studies compared existing models, six focused on the development of new PK-PD models, and two explored broader implications of TCI in anesthesia. Comparative studies indicate that while the Eleveld model generally offers superior predictive accuracy, it does not consistently outperform the Marsh and Schnider models across all populations. The Schnider model demonstrated better bias control in elderly patients, while the Eleveld model improved drug clearance estimation in obese patients. However, inconsistencies remain in predicting brain concentrations of propofol. Newly proposed models introduce adaptive dosing strategies, incorporating allometric scaling, lean body weight, and machine learning techniques, yet require further external validation. The results highlight ongoing challenges in achieving universal applicability of TCI models, underscoring the need for future research in refining precision dosing and personalized anesthesia management.

## Introduction

Target-controlled infusion (TCI) is an innovative medical therapy method that significantly changes how drugs are administered. TCI relies on pharmacokinetic models and algorithms to maintain a physician-defined target drug concentration in the patient’s plasma or effect site, allowing for reliable and consistent drug delivery. The infusion rate is dynamically adjusted to reach and maintain the desired concentration, thereby supporting the maintenance of a therapeutically effective drug level within the optimal range. Continuous clinical monitoring remains essential to ensure patient safety and to respond promptly to any physiological changes that may require intervention. This approach incorporates fundamental dose adjustments based on individual patient characteristics—such as age, body weight, sex, and other physiological parameters—through objectively defined functions embedded within the core pharmacokinetic and pharmacodynamic models. As a result, the extent of manual dose modifications required by clinicians to achieve individualized pharmacotherapy in a specific clinical context is considerably reduced [1].

TCI utilizes pharmacokinetic-pharmacodynamic (PK-PD) models to adjust the infusion rate based on population parameters. The pharmacokinetic (PK) model predicts the drug’s plasma concentration, while the pharmacodynamic (PD) model accounts for the delay between changes in plasma concentration and the clinical effect. Second-generation open-loop TCI systems incorporate the site of action into a three-compartment model, enabling better control of drug concentration in the brain through the ke₀ parameter. Modern closed-loop systems automatically adjust the infusion based on feedback from clinical parameters, ensuring more precise dosing, reducing the risk of hypotension, and improving postoperative recovery [2, 3].

With TCI, physicians can better control and tailor treatments to each patient’s needs, enhancing therapeutic effectiveness while potentially reducing the risk of side effects and treatment-related complications. Thus, TCI represents an important advancement toward individualized and safer medical care [4]. In 2018, the Association of Anaesthetists of Great Britain and Ireland, in collaboration with the Society of Intravenous Anaesthesia, published guidelines for the safe administration of total intravenous anesthesia (TIVA). The introduction outlines ten key recommendations, one of which states that when general anesthesia is maintained with a propofol infusion, the use of TCI is recommended [1].

Although TCI offers many advantages in clinical practice, concerns remain, particularly regarding the validity of existing mathematical models and their applicability in complex clinical scenarios [5]. However, despite advances and the inclusion of a wider range of covariates, available studies suggest that certain demographic groups remain underrepresented in its design [3, 6, 7]. This raises the question of whether a truly universal model can be developed—one that is fully applicable to all patient groups and accounts for variations in body weight, age, sex, and other physiological parameters. This review aims to evaluate the current state of research on PK-PD models for propofol TCI, identify studies highlighting the limitations of existing models, particularly regarding their ability to account for demographic variability, and outline key directions for future development.

## Methods

This systematic review was designed and conducted in accordance with the PRISMA 2020 guidelines (Preferred Reporting Items for Systematic Reviews and Meta-Analyses), and the systematic search followed the recommendations outlined in the PRISMA checklist [8]. PubMed, Summon, Google Scholar, Web of Science, and Scopus databases were systematically searched to identify relevant studies on the use of TCI for propofol administration. Key terms were combined using Boolean AND and OR operators to refine the search and focus on specific aspects of the research. Keywords included “target-controlled infusion AND models AND propofol” and “propofol AND (Marsh OR Schnider OR Eleveld).” This strategy ensured efficient retrieval of information related to pharmacokinetic and pharmacodynamic models for propofol.

The search was restricted to peer-reviewed articles published in English between January 1, 2020, and December 31, 2024, to provide an up-to-date perspective on the research topic. Inclusion criteria encompassed studies examining propofol administration via TCI in adult patients using the Marsh, Schnider, or Eleveld models, either in direct comparison or concerning other models derived from them. Exclusion criteria included studies on pediatric anesthesia, research focusing on anesthetics other than propofol (unless specifically comparing them with propofol), and papers lacking new data or consisting of major review articles already covered in previous studies.

Data collection from the selected studies was performed using systematic text analysis and key information extraction. A standardized data extraction form was developed to record general study details—such as title, authors, publication year, and country of origin—as well as specific study characteristics, including population descriptions and the types of models used. The extraction process focused on classifying mathematical models to identify the most commonly used ones, compare them, and evaluate their applicability to adult patient groups, as well as their role in simulations and the development of new models. Data were organized and analyzed using an MS Excel spreadsheet.

The study selection process began with the definition of inclusion and exclusion criteria, followed by a two-step systematic screening. Titles and abstracts were reviewed to exclude irrelevant studies, and full-text analysis was conducted for those meeting the entry criteria. The selection process was documented using a PRISMA flow diagram, which detailed the number of studies identified, included, and excluded, along with reasons for exclusion recorded in a summary table.

The synthesis results were presented in tables comparing different mathematical models and their applicability to various populations. A qualitative synthesis was used to analyze the data, summarizing and comparing key findings from individual studies, including the accuracy of prediction models, their robustness across different population groups, and comparisons of pharmacokinetic and pharmacodynamic parameters.

## Results

**Fig. 1:**
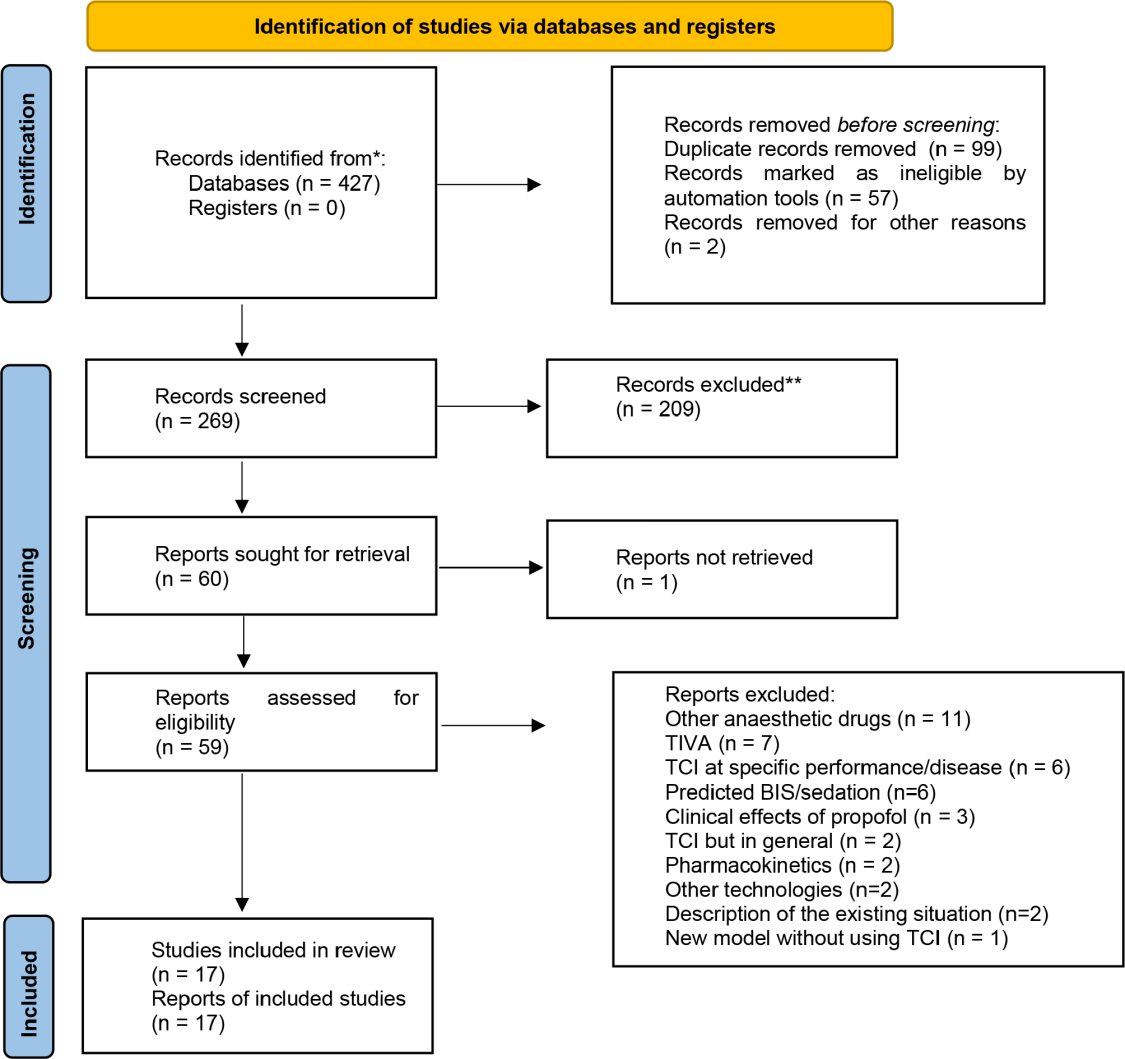
Systematic review flow diagram

A total of 427 sources were identified. After removing 2 non-English sources, 99 duplicates, and 57 sources flagged as inappropriate by automated tools (including letters, interim articles, editorials, corrections, and meeting abstracts), 269 relevant articles remained for further processing. Following a title and abstract screening, 209 articles were excluded for not focusing on anesthesia, propofol administration, or target-controlled infusion. This left 60 articles for detailed review, one of which was inaccessible due to a paywall. The full texts of the remaining 59 publications were systematically analyzed for relevance. The main exclusion criteria included studies using TCI for specific conditions or diseases, investigating drugs other than propofol, or focusing on general intravenous anesthesia. Ultimately, 17 articles were included in the study (See Fig. 1).

Seventeen studies on propofol TCI administration were identified, compared, and analyzed. Six studies focused on the development of new propofol models, nine compared existing models from different perspectives, and two were relevant to this work in terms of focus, timeliness, and results but did not fit into the previously defined categories.

Table 1 summarizes studies comparing existing TCI propofol models. The comparative studies by Hüppe et al. [9] and Linassi et al. [7] indicate that while the Eleveld model generally demonstrates better predictive performance, it does not consistently outperform the Marsh and Schnider models in all cases. Linassi et al. found that the Eleveld model had a higher incidence of adverse events compared to the Schnider model and exhibited greater bias in older patients.

In contrast, Hosseinirad et al. [10] reported that all three models performed equivalently in both open- and closed-loop systems, with no clear advantage among them. Vellinga et al. [11] prospectively validated the Eleveld model in a broader patient population, showing a predictive accuracy of < 30%, comparable to other models, confirming its suitability across different demographic groups.

Kim [12] focused on obese patients, highlighting that the Eleveld model improves drug delivery accuracy by better estimating clearance. Schnider et al. [13] investigated variability in CeT, finding that only 10.2% of the variability could be attributed to patient factors.

Van Hese et al. [14] examined differences between plasma and brain tissue concentrations of propofol and remifentanil. Their findings indicate that the Marsh model underestimates propofol concentrations, with brain concentrations exceeding plasma levels. Further research is needed to understand these discrepancies.

Wu et al. [15] examined pharmacodynamic responses to propofol administered via TCI using the Schnider model in patients with varying BMI. Although a shorter induction time was observed in patients with higher BMI, the study did not directly investigate the causal effect of BMI on induction time. Rather, the findings likely reflect the way BMI is incorporated into the Schnider model’s covariate structure, suggesting a model-driven outcome rather than a physiological association.

Coetzee et al. [16] compared the modified Marsh model with the Schnider model in terms of propofol concentration at the site of action. Their findings suggest that Ce-TCI with both models produces comparable hypnotic effects. However, a limitation of the study is its restricted generalizability, as the results likely apply only to young adults.

**Table 1:** Studies comparing existing models

Author, year	Study topic	Number of respondents	Monitored parameters	Results
Hüppe et al., 2020 [9]	Comparison of models Schnider, Marsh, and Eleveld	50	Arterial plasma concentration of propofol, MdAPE, MDPE, the ratio between calculated and actual plasma concentration	It has not been demonstrated that the Eleveld model provides better predictive performance for measured plasma concentrations; validation in an independent population is necessary.
Linassi et al., 2023 [7]	Comparison of models Schnider and Eleveld	78	BIS, CeP, LoR, MA, RoR, Procedure duration, anesthesia duration, time to RoR, total dose of propofol, number of adverse events	In the Eleveld model group, a higher incidence of adverse events was observed; the Eleveld model showed greater bias in MDPE among older patients.
Hosseinirad et al., 2024 [10]	Comparison of models Shuttler, Schnider, and Eleveld	/	Variability of PD models across three demographic groups, multiplicative uncertainty, established criteria and indices for comparing PD model variability and its impact on open-loop and closed-loop control	The models are equivalent in terms of applicability across different demographic groups in both open-loop and closed-loop systems.
Vellinga et al., 2021 [11]	Prospective validation of the Eleveld model; broader spectrum of patients	100 (4 × 25)	Arterial plasma concentrations of propofol, BIS, predictive performance of the model (MDPE, MdAPE), Ce	The predictive accuracy of the Eleveld PK/PD model was < 30%; it was not inferior to the others.
Kim, 2021 [12]	Comparison of models Schnider and Eleveld in obese patients	/	The volume of the central compartment, clearance	With relatively high clearance, a higher drug dose is required to maintain the same target concentration; the Eleveld model enables more precise drug administration.
Schnider et al., 2021 [13]	The impact of patient characteristics on variability CeT	4584	CeT, BIS	10.2% of the variability in CeT can be explained by patient factors; potential limitations of the study
Van Hese et al., 2020 [14]	Comparison of propofol and remifentanil concentrations in plasma and brain tissue, Marsh and Minto models	38	Predicted concentrations in plasma and at the site of action, sampling from the artery and brain tissue	The Marsh model demonstrated an overall underestimation of propofol concentrations, which were higher in the brain compared to plasma; further studies are needed.
Wu et al., (2022) [15]	Pharmacodynamic effects of propofol TCI using the Schnider model across different BMI categories	172	Induction time, induction dose, recovery time, Ramsay score, BIS, effect compartment concentration	Shorter induction time with higher BMI; differences likely reflect covariate structure of the Schnider model; further optimization is needed for diverse populations.
Coetzee et al., 2021 [16]	Comparison of adjusted-Mars model and Schnider model	40	BIS, time to LOC, Ce, effective k_e0_	Comparable hypnotic effects, clinically non-significant differences in BIS, faster k_e0_ - better precision targeting effect

Table 2 lists studies describing newly proposed models. The analysis of studies focusing on both PK and PD models for Propofol administration via TCI highlights the diversity of approaches and outcomes when comparing commercially used models, particularly Marsh, Schnider, and Eleveld.

**Table 2:** Studies of the proposed models

Author, year	Study topic	Number of respondents	Methods used	Results
Araújo et al., 2020 [17]	The new PK/PD model	60	BIS pro PD, Gas chromatography, NONMEM, Janmahatsu´s formula	Acceptable performance accuracy and the ability to directly adjust target BIS values concerning the predicted target concentration at the site of action
Braathen et al., 2024 [6]	The new universal PK model	69	UHPLC-MS/MS, NONMEM, PLT Tools, Allometric scaling	Lower bias and higher accuracy; validation of data on an external dataset is required; PD modeling is missing; potential limitations
Zhong a Xu, 2024 [18]	Changed model Eleveld	2768	Adjustment of input weight in the model Marsh, MATLAB	Good results up to 4 h of infusion; further testing is necessary
Kawata et al., 2024 [19]	The new PK model	57 (29/28)	NONMEM, k_e_ 0 0.014 min^-1^	A new k_p_ value enabling accurate description of higher concentrations in the brain; potential limitations; further testing is required
Kim et al., 2023 [20]	The new PK/PD model for elderly Korean patients	51 (31 for development/20 for validation)	BIS, NONMEM, validation by bootstrap, and predictive checks	Lower bias in the prediction of plasma propofol concentrations compared to the Eleveld; model is for older non-obese patients; limited use (45–75 kg) and validation in healthy volunteers only.
Paolino et al., 2024 [21]	Use of Eleveld model for PID control design	13 (induction phase) 300 (simulation on a large population)	BIS, MATLAB, boxplots, comparison of personalized vs. population control	Personalized PID controllers - more precise and stable control of anesthesia, aster achievement of the BIS target

Araújo et al. [17] and Braathen et al. [6] developed new PK-PD models using modern techniques such as allometric scaling and advanced analytical methods. While Braathen’s model demonstrated lower bias and higher accuracy, the need for further validation with external data underscores that these promising models still require additional testing. Zhong and Xu [18] modified the Marsh model to enhance its performance in short-term infusions. However, like other studies, their model requires further validation on external datasets.

In contrast, Kawata et al. [19] aimed to provide a more precise description of propofol concentration in the brain, representing a step toward optimizing models for specific clinical applications. However, the study acknowledged certain limitations, necessitating further testing. Similarly, the Choi model [20], developed specifically for elderly Korean patients, exhibited lower bias in plasma concentration predictions compared to the Eleveld model. Despite its promising performance, the Choi model is constrained by a narrow weight range (45–75 kg) and validation conducted only on healthy volunteers, highlighting the need for broader testing across diverse populations.

Paolino et al. [21] modified the Eleveld model by incorporating a PID controller that automatically adjusts the propofol infusion rate, enhancing anesthesia control. However, the authors note that the Eleveld PK-PD model was originally developed for pharmacokinetic prediction rather than regulatory purposes. Still, the personalized PID controller outperforms traditional approaches in achieving better anesthesia management.

Two additional studies, though not fitting into the previous categories, are noteworthy due to their focus and findings.

Vellinga et al. (2023) investigated the use of universal models for intravenous anesthetics, specifically propofol, and remifentanil, compared to traditional models. Developed using data from a diverse range of patient groups, these universal models reduce the risks of extrapolation and model misuse. Additionally, they simplify TCI administration by eliminating the need for anesthesiologists to fully understand the limitations of individual pharmacokinetic models, thereby reducing workload. This approach also minimizes the need for target concentration adjustments during the maintenance phase of anesthesia [22].

Schnider et al. examined the advantages and disadvantages of total intravenous anesthesia (TIVA) with TCI compared to inhalation anesthesia with EEG monitoring. TIVA with TCI facilitates automated drug titration, lowers the incidence of postoperative nausea and vomiting (PONV), and reduces healthcare workers’ exposure to anesthetic agents. Conversely, inhalation anesthesia offers better organ protection by reducing inflammatory responses and has demonstrated beneficial effects in septic patients. The authors also highlight the risks of relying on a single anesthetic agent, a concern underscored by propofol shortages during the COVID-19 pandemic. While TCI enables precise regulation of propofol concentrations and rapid adjustments to steady-state levels in response to changing clinical conditions, recent studies have questioned the benefits of TIVA over inhalation anesthesia, particularly regarding long-term mortality following cardiovascular surgery [23].

## Discussion

This systematic review demonstrates that while the Marsh, Schnider, and Eleveld models remain the most widely used in clinical TCI of propofol, none can be considered universally optimal across all patient populations and clinical scenarios. Each model has specific strengths and limitations depending on patient characteristics, clinical context, and the phase of anesthesia. Published studies on TCI for propofol primarily focus on comparing existing commercial models and developing new ones based on published designs. Currently, the Marsh, Schnider, and Eleveld models are among the most widely used. The Marsh model is the simplest, considering only body weight as a variable. The Schnider model is more advanced, incorporating additional covariates such as lean body mass (LBM). The Eleveld model, the most sophisticated to date, is based on the largest dataset and includes the most covariates, including allometric scaling [1–3].

An analysis of published studies reveals several inconsistencies, particularly regarding the number of patients used to define the Marsh model. This model was initially based on the Gepts model, which included three groups of six patients each [24]. Using these data, Marsh et al. published a 1991 study involving 20 children, which was later followed up by other researchers. However, different sources report varying patient numbers [25]. For example, Vandemoortele et al. [4] and Eleveld et al. [26] cite 37 patients, while Short et al. [27] mention 16, and Bidkar et al. [5] report as many as 150. These discrepancies highlight the lack of uniformity in data documentation and interpretation.

Absalom et al. [28] reported that the Marsh model is essentially identical to the Gepts model, except for an adjusted central compartment volume of 0.228 L/kg, though no detailed justification for this adjustment has been provided. Another point of confusion concerns the ke₀ value for the Marsh model. The original model used ke₀ = 0.26 min⁻¹, but the data supporting this value have not been published in peer-reviewed literature. However, Billard et al. reported a similar value of 0.2 min⁻¹. Currently, the so-called modified Marsh model is more commonly used, incorporating ke₀ = 1.21 min⁻¹ [28].

Coetzee et al. confirmed the effectiveness of this modification, demonstrating that a faster ke₀ improved the accuracy of targeting propofol’s effect to a level comparable to the Schnider model [16]. The ke₀ value is also a topic of discussion for the Schnider model, as it influences the balance between plasma and site-of-action concentrations. The Schnider model is commercially available in two variants: the SchniderK model, which has a fixed ke₀ value, and the SchniderT model, which features a variable equilibration constant but a fixed time-to-peak effect (TPE) [15].

After implementing the PK model in open TCI systems, an error was identified in the formula for calculating fat-free body mass. As weight and height increase, this value reaches a maximum before decreasing, potentially becoming negative. Consequently, clearance in obese individuals rises to unrealistic levels, as it is inversely proportional to muscle mass but dependent on weight and height. To address this issue, pump manufacturers modified the algorithm to ensure that fat-free body mass does not fall below its maximum [29].

Studies analyzing existing propofol models provide detailed insight into the predictive capabilities and limitations of current PK-PD models. Comparing these models across various clinical and experimental contexts highlights their strengths and weaknesses while identifying opportunities for further refinement.

Existing studies often compare pharmacokinetic models based on their ability to predict plasma and site-of-action concentrations of propofol. Regarding plasma concentration accuracy, the study by Hüppe et al. favors the Eleveld model, a finding also supported by Vellinga et al. However, when comparing how models represent the equilibration between plasma and brain concentrations, Linassi et al. highlight that the Schnider model assumes a higher ke₀ value. This parameter implies a more rapid effect-site equilibration, which mathematically reduces the size of bolus doses during target increases. In contrast, the Eleveld model demonstrates greater accuracy during the maintenance phase. The challenge of predicting brain propofol concentrations is also emphasized by Van Hese et al. [7, 9, 11, 14].

Another key area of interest is the role of input covariates. Wu et al. observed that the induction dose of propofol varied across BMI categories when using the Schnider model, with lower doses required in overweight and obese patients. These findings reflect how BMI-related covariates are incorporated in the model, rather than indicating a direct physiological effect of BMI. Kim et al. emphasize that incorporating covariates such as lean body weight (LBW) can greatly improve dose prediction in obese patients. In contrast, Schnider et al. report that only 10% of the variability in target propofol concentrations can be attributed to demographic factors like age or weight. The majority of this variability remains unexplained, underscoring the complexity of PK-PD processes [12, 13, 15].

Processed electroencephalogram (pEEG) monitoring, such as the Bispectral Index™ (BIS; Medtronic, Minneapolis, MN, USA), has been highlighted in several studies as a valuable tool for optimizing the depth of anesthesia and improving the clinical applicability of TCI systems. pEEG monitoring enhances pharmacodynamic precision and supports more individualized dosing strategies by providing real-time feedback on the patient’s hypnotic state [7, 10, 13].

In conclusion, the Eleveld model appears to be the most suitable for a broad patient population due to its extensive underlying database and high adaptability across age and body habitus. While the Schnider model is widely used and includes age as a covariate, current evidence does not support a clinically significant advantage in older patients regarding reducing anesthesia depth. Existing models continue to face limitations in accurately predicting brain propofol concentrations and addressing interindividual variability.

Future research should focus not only on integrating pharmacodynamic parameters—particularly brain concentrations—but also on clarifying the relationship between target concentrations and hemodynamic responses. While it is well established that hemodynamic effects influence the appropriate dosing of propofol, there is a lack of clinically applicable studies that directly address this interaction. Additionally, the development of advanced algorithms for adaptive dose management could further improve the safety and efficacy of targeted propofol infusion in diverse patient populations.

Comparative studies of existing models indicate that none is universally superior. While the Eleveld model often demonstrates higher accuracy, its performance is not consistently better in all situations. For example, it may exhibit greater bias in older patients, whereas the Schnider model is more effective in preventing adverse effects. The Marsh model is frequently criticized for underestimating propofol concentrations in brain tissue, potentially compromising prediction accuracy. In contrast, the Eleveld model more accurately estimates clearance in obese patients, which is crucial for precise dosing in this population.

Recent studies on optimizing TCI models demonstrate substantial progress, with five of the six analyzed studies published within the last two years. These works introduce innovative approaches to PK-PD modeling, enhancing dosing accuracy and personalization. The models proposed by Araújo, Braathen, Zhong, Kawata, and Choi address the limitations of existing models, such as Marsh, Schnider, and Eleveld, which have restricted applicability in certain patient populations.

Paolino et al. modified the Eleveld model by incorporating a personalized PID controller. Since the model was not originally designed for dynamic propofol administration, its use in closed-loop control requires modifications and has limitations, particularly in patients with prolonged pharmacodynamic delays. Nevertheless, the PID controller significantly improved the stability and accuracy of BIS control [21].

A significant challenge in TCI is addressing variability in age, body mass, and metabolism. Studies have explored this issue by examining the impact of weight on dosing accuracy. Araújo et al. emphasized the importance of using lean body weight (LBW) instead of total body weight (TBW), highlighting its critical role in achieving precise dosing. Similarly, Braathen et al. addressed the limitations of existing models, particularly the underrepresentation of patients with BMI > 50 kg/m² in the Eleveld model. To bridge this gap, they integrated both LBW and TBW alongside allometric scaling, enhancing applicability across diverse patient groups. Zhong et al. further contributed by refining weight input parameters in the Marsh model, improving its precision and usability [6, 17, 18].

Despite differing focal points, these studies share a common goal: enhancing predictive accuracy and reducing bias in propofol administration via TCI. Advanced methodologies, including allometric scaling, brain concentration analysis, and BIS monitoring, are crucial for developing more precise PK-PD models.

While Araújo and Braathen emphasize the importance of considering patient heterogeneity, particularly concerning age and body weight, Zhong and Kawata focus on refining pharmacokinetic predictions and brain-related parameters. Choi complements these efforts by proposing population-specific models tailored to the unique characteristics of defined patient groups. However, current evidence does not yet support a clear advantage of population-specific models over general-purpose models in broader clinical contexts, such as across different anesthesia techniques, co-medications, or centers. This highlights the ongoing need for external validation and careful assessment of model generalizability.

Newer models, such as those by Braathen and Choi, demonstrate reduced bias compared to commercially used models like Marsh, Schnider, and Eleveld. However, their applicability remains limited due to the need for extensive validation in larger population samples and external datasets. For instance, Choi’s model has only been tested on healthy volunteers within a narrow weight range, restricting its generalizability. Nevertheless, these approaches represent significant progress toward more personalized and effective propofol administration [6, 17–20]. A unifying feature of these studies is their use of advanced methods—including allometric scaling, brain concentration analysis, and BIS—to improve prediction accuracy and reduce bias in specific patient groups.

The final two articles analyzed underscore the importance of modern approaches to general anesthesia, TCI, and universal PK-PD models. Vellinga et al. focus on technological advances in TCI and their implications for clinical practice, while Schnider provides a broader perspective on the debate between TCI and inhalational anesthetics, examining the advantages and limitations of both approaches.

Vellinga et al. emphasize that universal models, developed using data from diverse populations, eliminate the need for manual parameter adjustments and reduce errors in marginal patient groups. This simplifies TCI application, enables safer and more effective dosing of anesthetics such as propofol and remifentanil, and improves clinical outcomes.

Schnider highlights additional benefits of TCI with propofol, including reduced postoperative nausea and vomiting (PONV), a lower risk of postoperative delirium, and more precise dose titration. Together, these advancements contribute to safer, more effective, and individualized patient care during anesthesia [22, 23].

Future development of TCI systems should prioritize adaptive closed-loop systems capable of responding in real time to changes in a patient’s physiological state. Integrating machine learning, BIS monitoring, and brain concentration modeling of propofol could significantly enhance dosing personalization and improve both the safety and efficacy of anesthesia.

Key priorities include validating these models in more diverse populations, incorporating universal models such as Eleveld into commercial infusion pumps, and utilizing multiparametric monitoring—combining BIS, hemodynamics, and drug concentrations—to more accurately control anesthesia depth.

## Conclusion

In conclusion, each existing model for target-controlled propofol infusion offers specific advantages but also has significant limitations that may lead to inaccuracies in pharmacokinetic and pharmacodynamic predictions, particularly in certain patient groups such as obese individuals, the elderly, or children.

Newly proposed models introduce innovative approaches by incorporating a broader range of input parameters, including allometric scaling, mass variable adjustments (e.g., LBW and TBW), standardized sedation depth measurements, and brain propofol concentration predictions. Despite promising results, a key challenge remains the extensive validation of these models across diverse patient populations to confirm their reliability and clinical efficacy.

## Data Availability

No datasets were generated or analysed during the current study.
